# Nowhere to hide: interrogating different metabolic parameters of *Plasmodium falciparum* gametocytes in a transmission blocking drug discovery pipeline towards malaria elimination

**DOI:** 10.1186/s12936-015-0718-z

**Published:** 2015-05-22

**Authors:** Janette Reader, Mariëtte Botha, Anjo Theron, Sonja B Lauterbach, Claire Rossouw, Dewaldt Engelbrecht, Melanie Wepener, Annél Smit, Didier Leroy, Dalu Mancama, Theresa L Coetzer, Lyn-Marie Birkholtz

**Affiliations:** Malaria Parasite Molecular Laboratory, Centre for Sustainable Malaria Control, Department of Biochemistry, University of Pretoria, Private Bag x20, Hatfield, Pretoria 0028 South Africa; Biosciences, Council for Scientific and Industrial Research, PO Box 395, Pretoria, 0001 South Africa; Plasmodium Molecular Research Unit, Wits Research Institute for Malaria, Department of Molecular Medicine and Haematology, School of Pathology, Faculty of Health Sciences, University of the Witwatersrand and National Health Laboratory Service, Johannesburg, 2193 South Africa; Medicines for Malaria Venture, Geneva, Switzerland

**Keywords:** Malaria, *Plasmodium falciparum*, Gametocyte, Gametocytocidal compounds, Transmission-blocking assays

## Abstract

**Background:**

The discovery of malaria transmission-blocking compounds is seen as key to malaria elimination strategies and gametocyte-screening platforms are critical filters to identify active molecules. However, unlike asexual parasite assays measuring parasite proliferation, greater variability in end-point readout exists between different gametocytocidal assays. This is compounded by difficulties in routinely producing viable, functional and stage-specific gametocyte populations. Here, a parallel evaluation of four assay platforms on the same gametocyte populations was performed for the first time. This allowed the direct comparison of the ability of different assay platforms to detect compounds with gametocytocidal activity and revealed caveats in some assay readouts that interrogate different parasite biological functions.

**Methods:**

Gametocytogenesis from *Plasmodium falciparum* (NF54) was optimized with a robust and standardized protocol. ATP, pLDH, luciferase reporter and PrestoBlue^®^ assays were compared in context of a set of 10 reference compounds. The assays were performed in parallel on the same gametocyte preparation (except for luciferase reporter lines) using the same drug preparations (48 h). The remaining parameters for each assay were all comparable.

**Results:**

A highly robust method for generating viable and functional gametocytes was developed and comprehensively validated resulting in an average gametocytaemia of 4 %. Subsequent parallel assays for gametocytocidal activity indicated that different assay platforms were not able to screen compounds with variant chemical scaffolds similarly. Luciferase reporter assays revealed that synchronized stage-specific gametocyte production is essential for drug discovery, as differential susceptibility in various gametocyte developmental populations is evident.

**Conclusions:**

With this study, the key parameters for assays aiming at testing the gametocytocidal activity of potential transmission blocking molecules against *Plasmodium* gametocytes were accurately dissected. This first and uniquely comparative study emphasizes differential effects seen with the use of different assay platforms interrogating variant biological systems. Whilst this data is informative from a biological perspective and may provide indications of the drug mode of action, it does highlight the care that must be taken when screening broad-diversity chemotypes with a single assay platform against gametocytes for which the biology is not clearly understood.

**Electronic supplementary material:**

The online version of this article (doi:10.1186/s12936-015-0718-z) contains supplementary material, which is available to authorized users.

## Background

Global efforts to eliminate malaria have achieved success in Europe and North America, but the disease remains a major health problem in sub-Saharan Africa [1]. Mortality rates have been reduced due to the deployment of insecticide-treated bed nets, indoor residual spraying and artemisinin-based combination therapy (ACT), but an estimated 198 million cases still occurred in 2013 [1]. One of the major realizations from previous elimination attempts is that, compared to smallpox and poliomyelitis, no single strategy will be applicable to control and eliminate malaria. Particularly evident is the fact that malaria elimination will not be achieved by focusing only on the treatment of the disease in humans or on vector control, but will require strategies to prevent transmission of the parasite between the human host and mosquito vector by targeting hepatic and gametocyte developmental stages [2, 3]. In 2007, the malaria eradication agenda was adopted by the global malaria community to galvanize coordinated efforts aimed not only to control malaria but also to eliminate it world-wide and ultimately eradicate this disease [1].

As pathogens, parasites of the genus *Plasmodium* have exquisitely adapted to varying biological environments in their human and mosquito hosts, with *Plasmodium falciparum* parasites causing the most virulent form of the disease. *Anopheles* mosquitoes introduce sporozoites into humans and these infect hepatocytes where, undetected, they replicate *en masse* and are released to initiate the pathogenic asexual cycle in human erythrocytes. Synchronized egress of merozoites from erythrocytes results in characteristic fever spells that typify the disease [4]. A portion of these *P. falciparum* parasites mature through five distinctive stages (I-V) into sexual forms in a process known as gametocytogenesis, lasting ~8 to 12 days, after which mature stage V male and female gametocytes are transmitted to mosquitoes for sexual reproduction [4, 5]. Mosquito uptake initiates important molecular and cellular changes in the gametocytes, enabling adjustment from the human to insect host. Contact with midgut factors activates the developmentally arrested gametocytes, resulting in egress from the erythrocyte, gamete formation and subsequent fertilization [6]. This is followed by the transformation of the fertilized zygote into the infective ookinete and oocyst that releases sporozoites for transmission back to humans [7].

Commitment to sexual development is postulated to take place in the first 20 h of the preceding erythrocytic cycle [8]. All merozoites from a single schizont will become either male or female gametocytes [9] with gender predetermined in the schizont committed to gametocytogenesis, but it is typically female-biased [10, 11]. Gametocyte production generally increases when the human host is anaemic with a low haematocrit and reticulocytosis [12].

Several population bottlenecks occur during the complete *Plasmodium* life cycle including the sexual developmental stages in the mosquito (e.g. only ~10 oocysts in mosquito midguts) as well as the hepatic sporozoite and intra-erythrocytic sexual gametocyte stages in humans. These, therefore, represent critical areas that could be successfully targeted for the ambitious goal of malaria elimination [13, 14]. However, given that a strategy to target the vector stages would require a drug to be at pharmacologically relevant concentrations for as long as mature gametocytes circulate (up to 30 days), the most appropriate point of intervention is to target the host gametocytes and eliminate the parasite population thus interrupting transmission. Currently, only artesunate, artemether and primaquine have therapeutic activity against late stage gametocytes, but these are threatened by emerging resistance and toxicity concerns (e.g. for primaquine in G6PD deficient patients) [15]. The development of new gametocytocidal compounds has therefore become a priority.

Standard mosquito membrane-feed assays remain the ultimate indicator of transmission-blocking ability of new compounds against *P. falciparum* parasites, but due to the highly demanding nature of this approach, gametocyte assays are key filters to identify transmission-blocking molecules [15]. However, there is a lack of standardization of methods to produce gametocytes and of assays to screen compounds. This makes it difficult to compare results from different laboratories and has hampered the discovery of new chemotherapeutics targeting gametocytes. The in vitro production of pure, viable, stage-specific *P. falciparum* gametocytes in high yield and in a consistent, reproducible manner for downstream screening assays is challenging and several methods have recently been published [15–21]. Gametocytogenesis was induced by nonspecific ‘stress’ to the parasite, including a drop in haematocrit [22], the use of ‘spent’ nutrient deficient culture medium [19], lymphocytes [23], mammalian hormones [24], reticulocytes [25], some inhibitors of nucleic acid synthesis (including antifolates) [22, 26, 27], Berenil, Fansidar, chloroquine, amodiaquine, sulphadoxine-pyrimethamine, ammonia compounds [28-30] and cholera toxin [12]. However, many of these methods are cumbersome, costly, not robust, and the gametocytes may not be suitable for evaluating potential transmission-blocking compounds.

Gametocyte screening assays should be reproducible and amenable to scaling up to medium- or high-throughput systems. Since gametocytocidal activity cannot be monitored with cellular multiplication markers, recent screens have typically relied on phenotypic assays. Systems developed thus far range from determination of gametocyte viability through detection of ATP [21, 31–33] and parasite lactate dehydrogenase (pLDH) [34, 35], colourimetric detection [31, 32, 36–38], flow cytometry [39] and transgenic reporter lines, which are useful for monitoring stage-specific effects [37, 40]. Some of these assays have been developed into reproducible medium- to high-throughput assays [38, 41, 42]. However, since these assays interrogate different metabolic pathways in the parasite, notable discrepancies in the outputs regarding the identification of compounds with gametocytocidal activity have been observed in the literature. Potential target compounds may therefore be missed if only one assay system is used. Direct comparison of assay platforms using a reference set of gametocytocidal compounds has not been reported yet. This could be highly informative to evaluate the robustness and validity of individual assay platforms in drug discovery programmes.

Here, a system for the production of gametocytes is reported that addresses several issues including: 1) increasing the number of parasites that commit to gametocytogenesis; 2) isolating large numbers of pure and viable parasites of a specific stage of gametocytes, and 3) reducing variability in gametocyte production, resulting in a robust system. Moreover, these gametocytes were subsequently used in a comparative study of four different assay systems on a single set of anti-malarial compounds provided by the Medicines for Malaria Venture (MMV). The ability of compounds to inhibit gametocytes was measured by detecting changes in the redox status of the parasite (modified alamarBlue^®^ assay [31, 41]), energy production (measurement of ATP levels [21, 32, 33, 36]), active glycolysis reflecting metabolic activity (pLDH [34]) and stage-specificity with a luciferase reporter assay [37]. Uniquely, all of the assays were performed on the same gametocyte population, with the same drug pressure applied for 48 h. This work enabled interrogation of the biological consequence of gametocytocidal compounds, irrespective of assay platform influences.

## Methods

### In vitro cultivation of asexual stage *Plasmodium falciparum* parasites

In vitro *P. falciparum* parasite cultures were maintained at 37 °C in human erythrocytes at a haematocrit of 5 % (ethics approvals: University of Pretoria 120821–077, CSIR Ref 10/2011, University of the Witwatersrand M130569) suspended in complete culture medium [RPMI 1640 medium (Sigma-Aldrich) supplemented with 25 mM HEPES (Sigma-Aldrich), 0.2 % D-glucose (Sigma-Aldrich), 200 μM hypoxanthine (Sigma-Aldrich), 0.2 % sodium bicarbonate, 24 μg/ml gentamicin (Invitrogen)] with either 0.5 % AlbuMAX^®^ II (Invitrogen) or 10 % human serum (Interstate Blood Bank, Chicago, USA) and flushed with 90 % N_2_, 5 % O_2_, and 5 % CO_2_ (Afrox, Johannesburg, South Africa) as described elsewhere [43, 44]. Medium was aspirated daily and replaced with fresh medium pre-warmed to 37 °C and parasite proliferation was monitored with microscopy of Giemsa-stained smears. Various *P. falciparum* strains (NF54, 3D7, FCR3, W2, HB3 and 7G8) were cultured in the same manner. In cases where asexual synchronicity was required, standard 5 % D-sorbitol synchronization was applied to ring-stage parasites [18].

### Induction of gametocytogenesis and maintenance of gametocyte cultures

This method was adapted from Carter et al. [45]. Gametocytogenesis was induced by a combination of nutrient starvation and a drop in haematocrit. Asexual parasites were cultured to a 6–10 % parasitaemia, which was then decreased to 0.5 % (at 6 % haematocrit) and the culture transferred to glucose-free medium. Cultures were maintained in an atmosphere of 5 % CO_2_, 5 % O_2_ and 90 % N_2_, at 37 °C, without shaking. Cultures were also kept at 37 °C during daily medium changes. After 72 h, the haematocrit was dropped to 3 % (day 0). Gametocytogenesis was subsequently monitored microscopically with daily medium (glucose-free) changes. On days 6–9, residual asexual parasites were eliminated by continuous 50 mM N-acetyl glucosamine (NAG), Sigma-Aldrich) treatment, now in the presence of 0.2 % glucose. Glucose enrichment was maintained from day 10 onwards and gametocytes monitored daily by microscopy until they were predominantly stage V and were used in the various assays.

### Male gamete exflagellation

Gametogenesis was assessed by treating samples with 50 μM xanthurenic acid (Sigma-Aldrich) in exflagellation buffer (RPMI 1640 with 25 mM HEPES, 0.2 % sodium bicarbonate, pH 8.0) followed by a >15 min incubation at room temperature. Exflagellation was visualized by phase contrast microscopy at 40x magnification.

### Validation of stage-specific gametocyte production

#### Flow cytometry and cell sorting

Flow cytometry was used to distinguish asexual parasites from different gametocyte populations. Gametocytes were enriched on CS-columns by magnetic separation in a VarioMACS magnetic system (Miltenyi Biotec) according to the manufacturer’s recommendations. Parasites were stained with Thiazole Orange (1 μM) for 20–30 min in the dark at 37 °C. Flow cytometry acquisition was performed for at least 50 000 events with a Beckman Coulter Gallios flow cytometer (TO: excitation at 488 nm; emission with 525/40 bandpass filter at 525 ± 20 nm). Gating of uninfected and infected erythrocytes was previously established and confirmed by Giemsa-stained microscopy of sorted populations. Uninfected erythrocytes were used as the negative control to determine background DNA fluorescence. Post-acquisition analyses were performed with Beckman Coulter Kaluza (v1.1) software.

#### Semi-quantitative RT-PCR

Real-time RT-PCR was used to confirm the gametocyte stages by evaluating differential transcript abundance in different gametocyte populations compared to asexual parasites, using primers for asexual and gametocyte-specific genes. Analysis was performed using the LightCycler^®^ 480 and KAPA SYBR Fast qPCR kit (Kapa Biosystems, USA) on 18 transcripts of interest (Primer sequences provided in Additional file 1: Table S1) using 5 pmol of each primer in 384-well plates; with cycling after pre-incubation at 95 °C for 10 min for 45 cycles (95 °C for 3 s, 55 °C for 7 s and 72 °C for 4 s). Relative expression was calculated using LightCycler^®^ 480 software (version 1.5) and the comparative C_T_ (2^-ΔΔCT^) method used to analyse transcript abundance.

### Gametocytocidal activity assays

#### Anti-malarial compounds/drugs

A 10-compound set was provided by the MMV, blinded until after data analysis. All other drugs were purchased from Sigma-Aldrich. Compounds were dissolved in DMSO and diluted fresh for each assay with glucose-rich complete culture medium to achieve a final concentration of 1 μM (final DMSO concentration ≤ 0.5 %). All assays were performed in parallel using the same stock of compounds, diluted fresh at the same time under the same conditions. Gametocytocidal activity was controlled by H_2_O_2_ (0.5 % continuous or 200 mM added at end-point) as well as drug controls, including methylene blue (1 μM) and dihydroartemisinin (DHA) (1 μM). Untreated gametocytes and uninfected red blood cells or culture medium were used to monitor viability and background, respectively.

#### Resazurin-based dye assay

The PrestoBlue^®^ (Life Technologies) assay was based on an adaptation of the method described by Tanaka and colleagues [31, 41]. Drug dilutions were placed in triplicate in 96-well plates in a volume of 50 μl/well. Semi-synchronous gametocyte cultures (50 μl/well, stage IV/V) were added to the 96-well plates to achieve a final gametocytaemia and haematocrit of 2 % and 5 % respectively, in a total incubation volume of 100 μl. The plate was placed on a shaker for 10–20 s before being encased in an air-tight chamber and gassed for 5 min with a 5 % CO_2_, 5 % O_2_, balance N_2_ mixture (Afrox, Johannesburg, South Africa). Following incubation at 37 °C for 48 h, 10 μl of PrestoBlue^®^ reagent was added to each well, the plate mixed on a shaker for 10–20 s and left to incubate at 37 °C for 2 h. Finally, the plate was centrifuged at 120xg (1 min), and 70 μl of the supernatant transferred to a clean 96-well plate before reading in a multiwell spectrophotometer (Infinite F500, Tecan, USA) by fluorescence detection at 612 nm.

#### pLDH assay

Drug dilutions were placed in triplicate in 96-well plates in a volume of 100 μl/well. Semi-synchronous stage IV and V gametocyte cultures (100 μl/well) were added to the 96-well plates to achieve a final gametocytaemia and haematocrit of 2 % and 1 % respectively, in a total incubation volume of 200 μl. The plates were incubated at 37 °C for 48 h. Gametocyte viability was determined spectrophotometrically by measuring the activity of pLDH [34], according to a modified version of the method of Makler and Hinrichs [46]. Briefly, 100 μl of Malstat reagent (0.21 % *v/v* Triton-100; 222 mM L-(+)-lactic acid; 54.5 mM Tris; 0.166 mM 3-acetylpyridine adenine dinucleotide (APAD; Sigma-Aldrich); adjusted to pH 9 with 1 M NaOH) was transferred into a clean 96-well plate. A fixed volume of 20 μl parasite suspension/well was added to the Malstat plate, followed by the addition of 25 μl PES/NBT (1.96 mM nitro blue tetrazoliumchloride NBT; 0.239 mM phenazine ethosulphate PES). Absorbance was measured with a Multiskan Ascent 354 multiplate scanner (Thermo Labsystems, Finland) at 620 nm.

#### ATP bioluminescence assay

Mature gametocytes, predominantly stage V, were enriched using density gradient centrifugation and magnetic separation. For density gradient separation, gametocytes were pelleted and resuspended in 10 ml of glucose-rich complete medium, loaded onto 5 ml preheated (37 °C) NycoPrep™ 1.077 cushions (Axis-Shield) and centrifuged at 800xg for 20 min at 37 °C. The gametocyte-containing bands were collected, concentrated by centrifugation and the pellet resuspended in 5 ml glucose-rich complete medium. This was loaded on equilibrated LS-columns for magnetic separation in a MidiMACS magnetic system (Miltenyi Biotec) to purify and enrich the gametocytes, which were counted in a Neubauer chamber. Drug dilutions were placed in triplicate in 96-well plates. Approximately 30,000 gametocytes in glucose-rich complete medium were added to each well in a final volume of 100 μl and the plates incubated for 48 h in a humidified gas chamber (90 % N_2_, 5 % O_2_, and 5 % CO_2_) at 37 °C. Subsequently, the BacTiter-Glo™ assay (Promega) was performed according to the manufacturer’s instructions at room temperature in the dark, with assay substrate incubation for 10 min and shaking for the first two minutes, to detect ATP levels. Bioluminescence [17, 21, 32] was detected at an integration constant of 0.5 s with the GloMax^®^-Multi + Detection System with Instinct^®^ Software.

#### Luciferase reporter assay

The Luciferase reporter assay was established to enable accurate, reliable and quantifiable investigations of the stage-specific action of gametocytocidal compounds for each of the early and late gametocyte marker cell lines; NF54-PfS16-GFP-Luc and NF54-Mal8p1.16-GFP-Luc (kind gift from David Fidock, Columbia University, USA) [37]. Gametocytogenesis was induced on synchronized asexual parasites as described above, with the exception that NAG treatment was initiated from day 1 – 4 to remove asexual parasites early and allow more synchronized early stage gametocytes. Drug assays were set up on day 5 and 10 (representing early stage I/II/III and mature stage IV/V gametocytes, respectively). In each instance, assays were set up in triplicate using a 2 % gametocytaemia, 2 % haematocrit culture and 48 h drug pressure in a gas chamber (90 % N_2_, 5 % O_2_, and 5 % CO_2_) at 37 °C. Luciferase activity was determined in 20 μl parasite lysates by adding 50 μl luciferin substrate (Promega Luciferase Assay System) at room temperature and detection of resultant bioluminescence at an integration constant of 10 s with the GloMax^®^-Multi + Detection System with Instinct^®^ Software.

### Data analysis

Assay quality was measured for all assays by defining the standard deviation (SD); standard error of the mean (SEM); percent coefficient of variation (%CV); signal to background ratio (S/B), signal to noise ratio (S/N) and the Z’-factor, which were calculated according to the formulae below [34]. The Z’-factor is a measure of statistical effect size. The result is given as a numerical value (0 to 1), being more favourable as it approaches 1 [32, 47]. %CV is acceptable at <20 % [48]. All parameters were calculated from at least three independent experiments for each assay, each time performed in triplicate. Results were expressed as the percentage inhibition compared to untreated controls.

$$ \mathrm{Z}'=1-\left[3\left({\mathrm{SD}}_{\mathrm{positive}} + {\mathrm{SD}}_{\mathrm{background}}\right)/{\mathrm{Mean}}_{\mathrm{positive}}\hbox{--}\ {\mathrm{Mean}}_{\mathrm{background}}\right)\Big] $$

$$ \mathrm{S}/\mathrm{N}=\left[\left({\mathrm{Mean}}_{\mathrm{positive}}\hbox{--}\ {\mathrm{Mean}}_{\mathrm{background}}\right)/{\mathrm{SD}}_{\mathrm{background}}\right] $$

$$ \mathrm{S}/\mathrm{B}={\mathrm{Mean}}_{\mathrm{positive}}/\ {\mathrm{Mean}}_{\mathrm{background}} $$

$$ \%\mathrm{C}\mathrm{V}=\left(\mathrm{S}\mathrm{D}/\mathrm{Mean}\right)\mathrm{x}\ 100 $$

$$ \mathrm{Percentage}\ \mathrm{gametocyte}\ \mathrm{viability}\ \left(\%\ \mathrm{Viability}\right)=\kern0.5em \left[\left(\mathrm{Signal}\ \hbox{--}\ {\mathrm{Mean}}_{\mathrm{background}}\right)/{\mathrm{Mean}}_{\mathrm{positive}}\hbox{--}\ {\mathrm{Mean}}_{\mathrm{background}}\right)\Big]\ \mathrm{x}\ 100 $$

$$ \%\mathrm{Inhibition}=100\hbox{--} \left(\%\mathrm{viability}\right) $$

## Results

### Optimization of *P. falciparum* gametocyte production

Various parameters were investigated for their ability to influence gametocyte production including: 1) the influence of stationary versus shaking culturing conditions; 2) AlbuMAX^®^ II *vs* human serum in culture medium; 3) evaluating different methods for removal of asexual parasites; and 4) evaluating strain-specific differences in gametocyte production (Table 1). This resulted in the following optimized protocol.

**Table 1:** Induction of in vitro gametocytogenesis in P. falciparum parasites under various conditions

Conditions		Gametocytaemia^a^	Conversion Factor^b^
Shaking vs. stationary (serum medium)			
Asexual culture	Gametocyte culture		
Stationary	Stationary	1.3 ± 0.6 % (*n* = 4)	15.8 ± 0.6 %
Shaking	Stationary	3.3 ± 1.4 % (*n* = 22)	34.2 ± 3.4 %
Medium composition (asexual, stationary gametocyte cultures)			
Asexual culture	Gametocyte culture		
AlbuMAX	AlbuMAX	5.1 ± 1.0 % (*n* = 10)	33.3 ± 1.9 %
	Serum	1.9 ± 0.8 % (*n* = 3)	8.2 ± 2.2 %
Serum	AlbuMAX	3.7 ± 1 (*n* = 3)	22.7 ± 1.1 %
	Serum	1.3 ± 0.6 % (*n* = 4)	20.7 ± 3.3 %
Asexual parasite elimination			
Using sorbitol		5.3 ± 0.2 % (*n* = 2)	32.3 ± 1.4 %
Using NAG		4.9 ± 1.0 % (*n* = 2)	37.7 ± 1.7 %
Different strains of *P. falciparum* parasites			
NF54		4.9 ± 1.0 % (*n* = 3)	35.0 ± 1.7 %
3D7		1.5 ± 0.2 % (*n* = 3)	11.2 ± 2.0 %
W2		1.1 ± 0.2 % (*n* = 3)	8.9 ± 0.5 %
7G8		1 ± 0.7 % (*n* = 2)	ND
FCR3		0 % (*n* = 3)	NA
HB3		0 % (*n* = 3)	NA

*ND* not detected / determined, *NA* not applicable

^a^ Day 11 after induction

^b^ $$ \mathrm{Conversion}\ \mathrm{factor}=\kern0.5em \frac{\mathrm{Number}\ \mathrm{of}\ \mathrm{stage}\ 2\ \mathrm{gametocytes}\ \mathrm{on}\ \mathrm{day}\ 4}{\mathrm{Number}\ \mathrm{of}\ \mathrm{rings}\ \mathrm{on}\ \mathrm{day}\ 2} $$

Data are representative of (n) biological experiments, each performed in triplicate, ± SEM. Data indicate maximal levels of gametocytaemia obtained

**Fig. 1:**
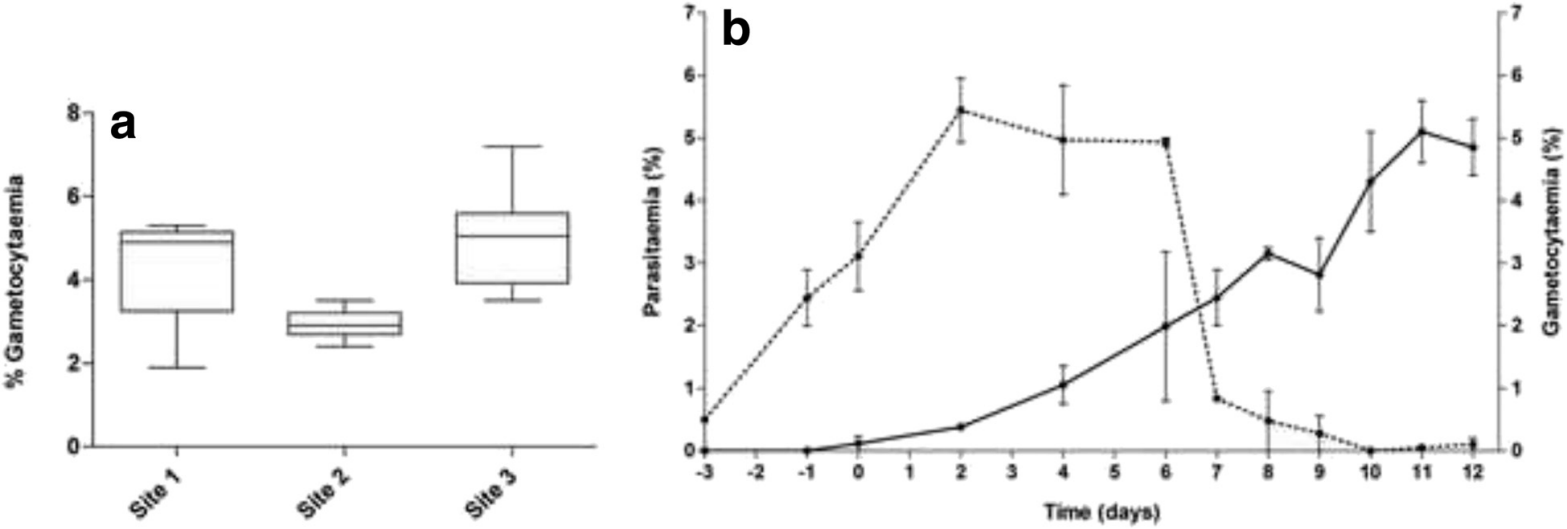
Evaluation of *P. falciparum* gametocyte production. Parasites (NF54 *P. falciparum*) were cultured in medium with 0.5 % AlbuMAX for both asexual and gametocyte cultures. **a** Box and whisker plots of gametocytaemia (% stage III-V gametocytes) obtained with the same optimized protocol across three independent sites (site 1 *n* = 6; site 2 *n* = 17; site 3 *n* = 14). **b** Kinetics of parasitaemia (asexual stages; dashed line) and gametocytaemia (gametocytes; solid line) during gametocytogenesis. The number of asexual forms increased up to maximum parasitaemia on days 2–6 (prior to NAG treatment). Sexual forms were first detected on day 2, with gametocytaemia reaching an average of 5 % on day 11. Data are from ≥4 independent biological experiments each performed in triplicate, ± SEM

Physiological stress was applied to a starting asexual culture by increasing parasitaemia to 6–10 % in a parasite population of >80 % rings. Gametocytogenesis was subsequently induced by a combination of both nutrient starvation (glucose deprivation) and a decrease in haematocrit under stationary culturing conditions in the presence of AlbuMAX^®^ II and gentamicin, whilst maintaining a 37 °C environment throughout (Table 1). Gametocytogenesis required growth under stationary conditions, from asexual cultures grown either under stationary or shaking conditions. Once gametocytes were observed microscopically, asexual parasites were removed from a predominantly stage II gametocyte population by applying NAG pressure for at least two asexual developmental cycles (from day 6–9), whilst maintaining gametocyte viability in a nutrient rich environment by adding glucose. This optimized protocol produced an average gametocytaemia of 5.1 ± 1.0 % on day 11. Moreover, a gametocyte conversion factor of 33.3 ± 1.9 % was obtained, indicating a high proportion of asexual parasites committed to gametocytogenesis (Table 1). This procedure was moreover highly robust as validated across three independent laboratories (Fig. 1a) that generated high quality, viable gametocytes in a high average yield of 3.9 ± 0.2 %.

The addition of the broad-spectrum gentamicin to the culture medium as preservative did not influence gametocyte production or viability (normal exflagellation and ATP production; Additional gametocyte viability indicators are provided in Additional file 2: Figure S1). However, gentamicin might influence downstream anti-malarial assays, particularly in the context of screening unknown test compounds. To investigate gentamycin interference, the viability of mature stage IV-V gametocytes grown in the presence and absence of gentamicin was determined after treatment with dihydroartemisinin (DHA). Treatment in the presence of gentamicin revealed the expected dose–response indicating no detrimental effect of gentamicin under these conditions and for this compound. Gentamicin was therefore included in the culture medium (Additional gametocyte viability indicators are provided in Additional file 2: Figure S1).

The kinetics of conversion to gametocytes followed the expected maturation to stage IV-V gametocytes achieved at day 10–12 (Fig. 1b). Asexual parasitaemia peaked at day 2 at ~5 %, where after this rapidly decreased on day 7 after NAG pressure. Sexual forms were visible from day 2 onwards, with peak gametocytaemia of ~5 % obtained on day 11 (Fig. 1b). The viability of parasites produced on the optimized protocol was confirmed with an ATP assay (Additional gametocyte viability indicators are provided in Additional file 2: Figure S2).

As previously reported, gametocytogenesis is strain dependent and this held true for the strains that were tested, where the ability to produce gametocytes varied under the same optimized culture conditions [19]. Of the *P. falciparum* strains tested, drug sensitive NF54 parasites remained superior in producing gametocytes, with the chloroquine-resistant W2 strain, the chloroquine-sensitive and sulphadoxine-resistant 3D7 strain, and the chloroquine- and antifolate-resistant 7G8 strain producing low levels of gametocytes (Table 1). No detectable gametocytes were observed in the pyrimethamine resistant HB3 strain and in the FCR3 strain, which is resistant to chloroquine and cycloguanil (Table 1). Maximal gametocytogenesis (1.5 % gametocytaemia, 11 % conversion factor) could only be achieved in freshly thawed 3D7 parasites and this decreased as parasites were kept in routine culture for more than 7 generations to 0.1 % gametocytaemia (~4 % conversion rate). For optimal gametocyte production on all strains, gametocytes were induced from freshly thawed asexual cultures within 2–3 generations after thawing but never from asexual parasites maintained for more than 6–9 generations.

### Validation of stage-specific gametocyte production

**Fig. 2:**
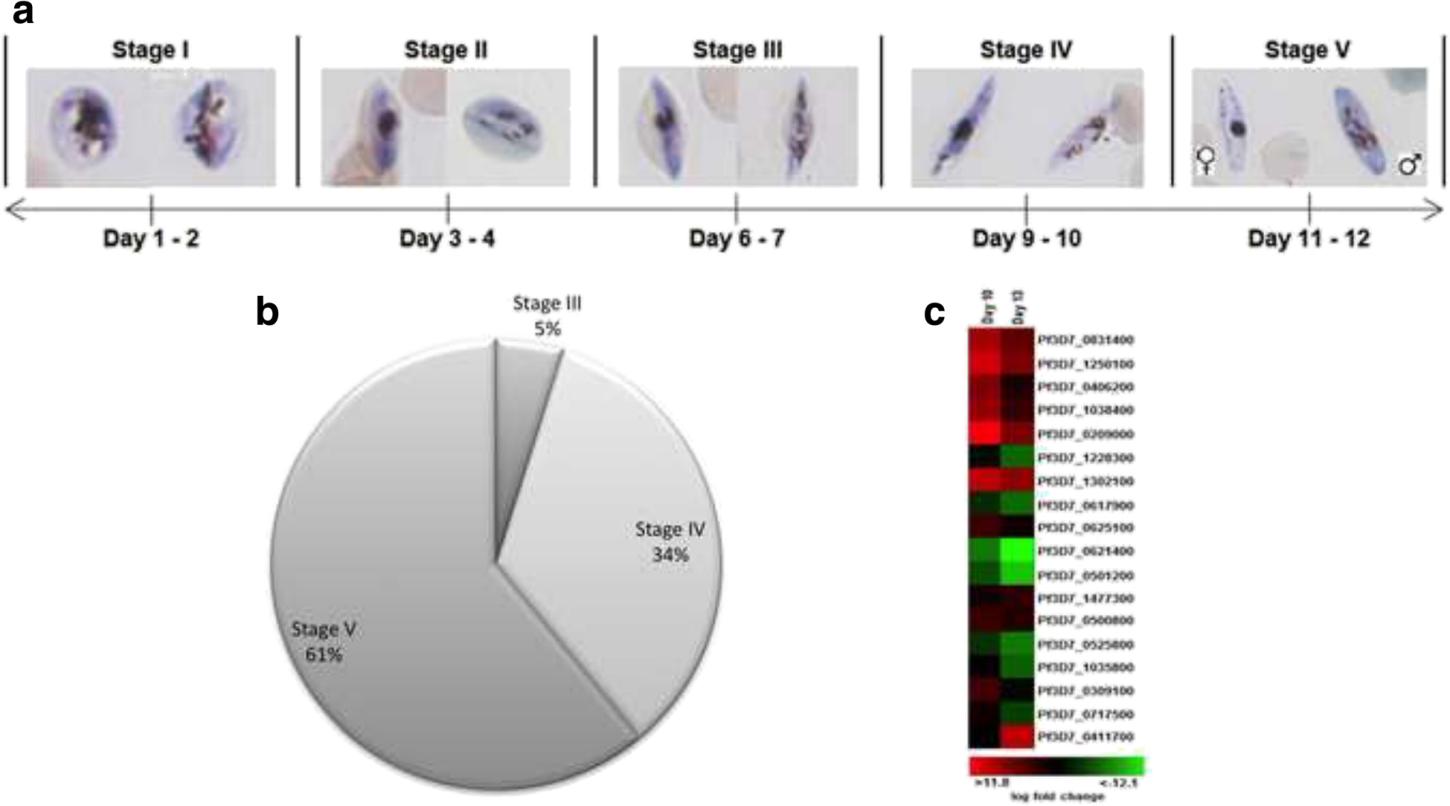
Stage-specific, quantitative analysis of gametocyte populations. **a** Giemsa-stained smears indicating morphology of different stages. **b** Stage distribution of gametocyte populations for 22 independent gametocyte cultures. **c** Semi-quantitative RT-PCR of stage-specific expression of 18 unique descriptors. PCR was performed and data normalized to cyclophillin as household expression control and expressed as fold change relative to background expression of the transcripts in asexual parasites. Data are from ≥4 independent biological experiments each performed in triplicate, from which expression ratios were determined. Under-expressed transcripts are presented in green and over-expressed transcripts in red

The production of gametocytes, synchronized to a small stage-specific window, is important in gametocytocidal drug discovery efforts, as evidence indicates differential susceptibility of gametocytes in different stages of development towards a variety of compounds [37, 38]. Stage-specific gametocyte production was ensured through the use of synchronized (>80 % rings) starting populations of asexual parasites and the application of NAG pressure at defined time points during gametocytogenesis. Developmental stages of gametocytes were evaluated morphologically based on the description of Hawking, 1971 (Fig. 2a) [49]. Synchronicity was quantified to >91 % mature stage IV/V gametocytes (91 ± 18 %, n ≥ 6) with <10 % contaminating early stage (II and III) gametocytes (Fig. 2b). This synchronicity was validated with semi-quantitative PCR based on the expression of certain transcripts in a stage-specific manner during gametocytogenesis [50]. The relative expression of a subset of 18 unique transcripts, chosen for their presence in early vs. mature gametocytes, was monitored during different stages of gametocyte development and compared to the expression levels of these transcripts in asexual parasites as an expression background (Fig. 2c). This allowed differentiation between gametocyte stages, particularly also allowing distinction and quantification of stage IV gametocytes from their mature stage V partners, where certain transcripts (e.g. PF3D7_0621400) are strongly activated and expressed in stage IV parasites, but not so in stage V parasites compared to others solely expressed in stage V parasites (e.g. PF3D7_0411700). These data could be informative regarding the stage-specific delineation of drug action and could allow investigations of the kinetics of drug action enabling therapeutic window of susceptibility descriptors.

**Fig. 3:**
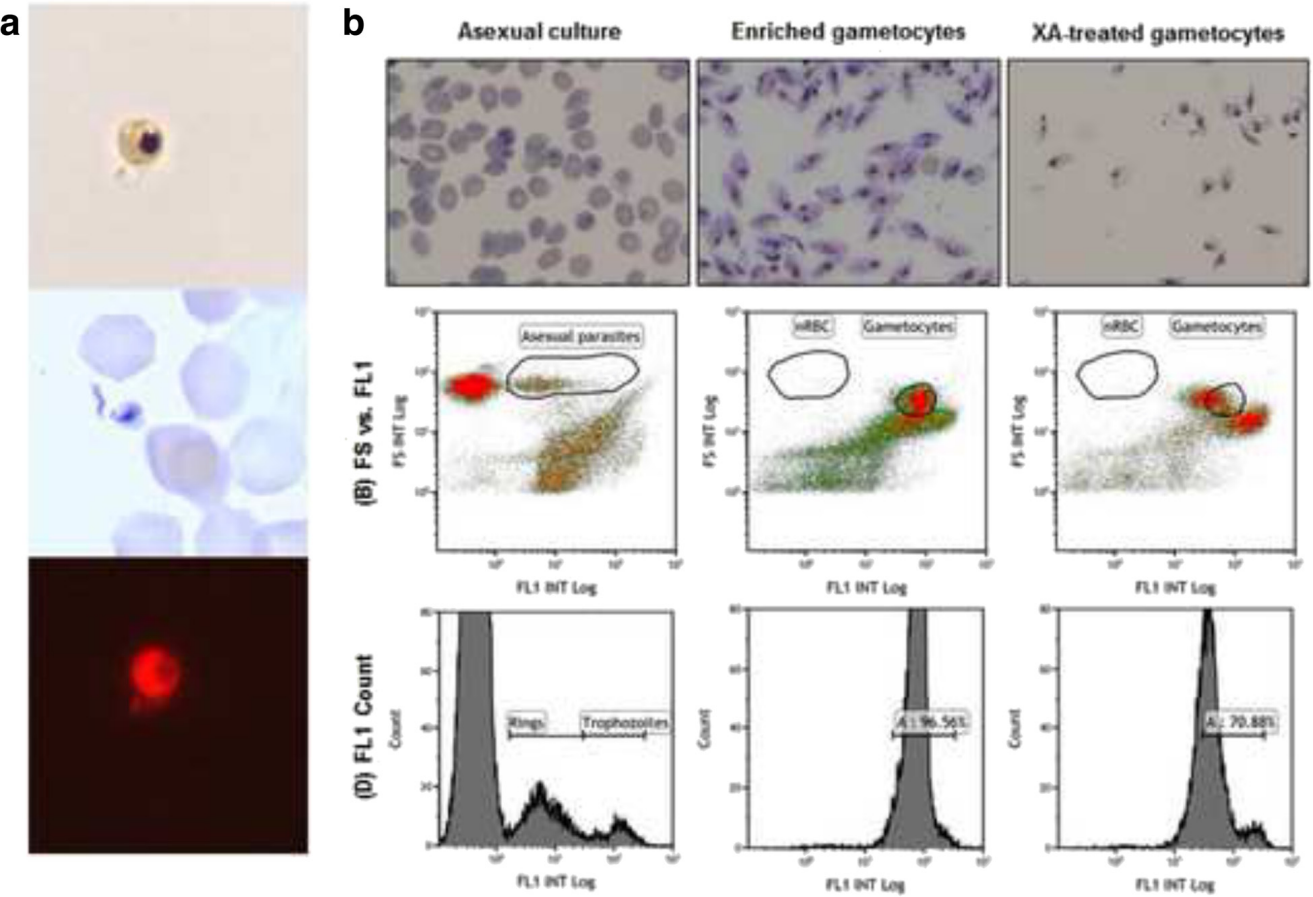
Functionality and viability of gametocytes. **a** Male exflagellation. White-light, Giemsa and Phalloidin stained real-time imaging of exflagellating microgamete. Visualized at 200x magnification. **b** Flow cytometric evaluation of asexual parasites and gametocytes by thiazole orange nuclear marker analysis. Gametocytes were purified on magnetic columns and treated with xanthurenic acid (XA) to induce exflagellation of male gametes

The viability and functionality of the gametocyte populations were confirmed by real time microscopic observation of male gamete exflagellation induced by xanthurenic acid and a decrease in temperature (Fig. 3a). Moreover, the mature gametocyte populations produced mimicked the in vivo female bias [8] with a 4:1 ratio of female:male gametocytes produced, as evaluated morphologically.

Flow cytometry was used as explorative technology to differentiate and quantify gametocyte populations. Asexual parasites were easily distinguishable from gametocytes and additionally various gametocyte populations (stages) could be identified based on their morphological and density differences (Fig. 3b). Subsequent gating of various populations within the enriched gametocyte sample allowed cellular sorting of between 100,000 and 5,000,000 cells. Samples were analysed microscopically to confirm identity of the suggested populations and discriminate between asexual and gametocyte populations. Additionally, to distinguish the mature male and female gametocytes, ~500,000 cells were treated with xanthurenic acid to induce gametogenesis. This produced two separate gamete populations and in this manner, clear gamete populations were distinguishable (Fig. 3b). Although informative from a qualitative point of view regarding quality control for gametocyte production, the flow cytometric method is however not amenable to continuous application in a screening programme.

### Assays for gametocytocidal activity

Four different gametocytocidal screening assays were evaluated and compared on mature stage IV-V gametocytes. The assays were chosen based on their ability to detect different biological activities as indicators of parasite viability. The bioluminescent ATP assay reflects energy status; pLDH is an indicator of active glycolysis; the colourimetric resazurin dye is a redox indicator and the luciferase reporter assays (under control of gametocytogenesis-specific promoters for *Pfs16* and mal8p1.16) determine stage-specific action of gametocytocidal compounds. Assay performance was evaluated for linearity, detection range, reproducibility and the assay screening window coefficient (Z’-factor) [48] and inter-assay reproducibility via %CV [51]. Moreover, drug incubation time (24 or 48 h), optimal haematocrit and optimal gametocytaemia were determined for each assay platform.

### ATP assay as indicator of parasite viability

The bioluminescent ATP assay measured luciferin-luciferase as an indicator of parasite viability, directly correlating ATP levels to luminescence output. Because ATP is present in erythrocytes, the assay requires almost 100 % pure gametocytes that are free of contaminating, uninfected red cells. The assay proved to be linear over the range of 4000 – 62,000 purified gametocytes (R^2^ = 0.99). S/N and S/B were high at >15,000 fold and >500 fold, respectively (Gametocytocidal assay metrics provided in Additional file 3: Figure S3). The assay was routinely performed on 30,000 gametocytes and 24 h drug treatment. Mature stage IV-V gametocytes were successfully enriched to >95 % purity with yields of ~677,500 ± 83,815 gametocytes per ml original culture volume (*n* = 4). The assay incubation time was monitored, with a dramatic decrease in ATP levels observed directly after enrichment, which then stabilized around 16–24 h. However, an average decrease of ~50 % was seen in ATP levels of untreated parasites between 24 and 48 h, with linearity decreasing from R^2^ 0.99 to 0.85 at 48 h. The assay produced good intra-assay variability with average Z’-factors of 0.84 and 0.79 at 24 and 48 h, respectively. However, a relatively high average %CV of 8.8 % was observed.

### pLDH assay as indicator of metabolic activity in viable parasites

The pLDH assay relies on the rapid utilization of 3-acetylpyridine adenine dinucleotide (APAD) as a coenzyme by *P. falciparum* lactate dehydrogenase in the reaction leading to the conversion of lactate to pyruvate [35, 46]. The pLDH assay revealed acceptable linearity profiles of R^2^ = 0.95 and 0.96 achieved at 0.5 % and 1 % haematocrit, respectively (Gametocytocidal assay metrics provided in Additional file 3: Figure S4). At 24 h, the Z’-factor, S/N and S/B ratios were markedly less than after 48 h of incubation (S/N of 28 vs. 300 at 24 vs. 48 h, respectively, Table 2). Z’-factor values decreased with decreasing gametocytaemia and haematocrit with the optimal gametocytaemia and haematocrit for the pLDH assay determined as 4.5 % gametocytaemia and 0.5 % haematocrit, based on a Z’-factor of 0.90. However, due to the typical gametocytaemia produced without enrichment, the assay was optimized to 2 % gametocytaemia and 1 % haematocrit with an acceptable Z’-factor of 0.87 attained at 2.3 % gametocytaemia and 0.5 % haematocrit. Average %CV was 2.4 % between independent experiments for the control compounds.

**Table 2:** Performance indicators of various gametocytocidal assay platforms

Assay performance parameters (avg)	ATP	pLDH	PrestoBlue^®^	Luciferase
	(*n* = 4)	(*n* = 4)	(*n* = 4)	(*n* = 4)
S/N	>15 000	>300	>900	16 000 (early)
				500 (late)
S/B	500	3.2	15	175
Z’-factor (avg)	0.79	0.87	0.91	0.81
%CV (intra-assay)	8.8 %	2.4 %	3.15 %	0.73 %
Avg DHA activity at 1 μM (% inhibition)	37.19 ± 7.67 %	62 ± 6 %	83.48 ± 8.58 %	73.56 ± 5.58 % (avg early/late)
IC_50_ DHA	14.9 μM^a^	20 nM	11 nM	43 nM (early)
				11 nM (late)
IC_50_ MB	900 nM^a^	800 nM	-	195 nM (early)
				143 nM (late)

*DHA* Dihydroartemisinin, *MB* Methylene blue

^a^unpaired experiments at 24 h

Each assay was performed after 48 h drug exposure and comparative quality control parameters determined, utilising standardization of DHA activity as common factor between all the assay platforms. Methylene blue interferes with the PrestoBlue^®^ assay and was not used as control. Data are from 4 independent experiments (performed in triplicate) for each assay platform

### PrestoBlue^®^ assay as an indicator of parasite respiration

PrestoBlue^®^ is a colourimetric reagent whose spectral features reflect the metabolic activity of cells. It is based on resazurin, a cell-permeant blue dye, which functions as a cell viability indicator when reduced to resorufin (red) by the metabolic activity of living cells. Resazurin reduction correlates almost perfectly with cellular respiration. Resorufin is around 12 times more fluorescent than resazurin and has an emission maximum of 584 nm [52, 53]. The colourimetric assay displayed a good linearity profile in the absence or presence of the drugs tested (R^2^ = 0.97) using a 2 % gametocytaemia and 5 % haematocrit. Upper and lower detection limits were estimated to be 97 % and 21 %, respectively, and the average %CV between independent experiments was estimated to be 3.15 % (Gametocytocidal assay metrics provided in Additional file 3: Figure S5). On average, the Z’-factor was estimated to be 0.91 (Table 2). Modifying the final gametocytaemia (using 0.5 %, 1 %, or 3 %), or by decreasing the drug incubation duration (24 h instead of 48 h) led to a general reduction in assay performance as determined by the Z’-factor.

### Luciferase reporter assay as an indicator of stage-specific gametocyte gene expression

Two transgenic parasite lines were employed in the luciferase assays viz. NF54-PfS16-GFP-Luc and NF54-Mal8p1.16-GFP-Luc [37], expressing a green fluorescent protein (GFP)-luciferase fusion reporter gene under the control of two gametocytogenesis-specific genes, *Pfs16* NF54_Pfs16_ luciferase activity peaks on day 2 of gametocytogenesis and then gradually declines [54]. Mal8p1.16 reporter expression in NF54_mal8p1.16_ is specific for late stage gametocytes, increasing only at day 6–8 and peaking at day 10–12 after gametocytogenesis [37].

These lines accurately and reproducibly allow detection and quantification of early (stage I/II) gametocytes from mature gametocytes (stage IV/V) [37]. Luciferase activity was determined for a stage I/II culture (day 5; 89 % stage I and II; 11 % stage III; *n* = 6) as well as mature stage IV/V gametocytes (day 11; 91 % stage IV/V; 9 % stage III; *n* = 6). This revealed quantitative evaluation of the stage distribution of gametocyte populations, independent of haematocrit interference (Gametocytocidal assay metrics provided in Additional file 3: Figure S6). This assay exhibited good intra-assay reproducibility (Z’-factor of 0.77 and 0.83 on average for early and late gametocyte markers, average of 0.81) as well as high sensitivity (S/N >500 or >16 000 for the late and early marker assays, respectively) and a %CV of 0.73 % (Table 2).

### Assay comparisons for gametocytocidal screens

The different assay platforms were also evaluated and compared for their ability to accurately determine IC_50_ of known gametocytocidal compounds DHA and methylene blue. Late stage gametocytocidal potency of DHA has been reported to be in the range of 2–26 nM whereas methylene blue potency is poorer, ranging between 12 and 490 nM [15, 38]. The pLDH, luciferase reporter and PrestoBlue^®^ assays all reported comparible low nM activities for DHA whereas the luciferase reporter assay seemed more sensitive to determine methylene blue IC_50_ compared to the pLDH and ATP assays (Table 2).

**Fig. 4:**
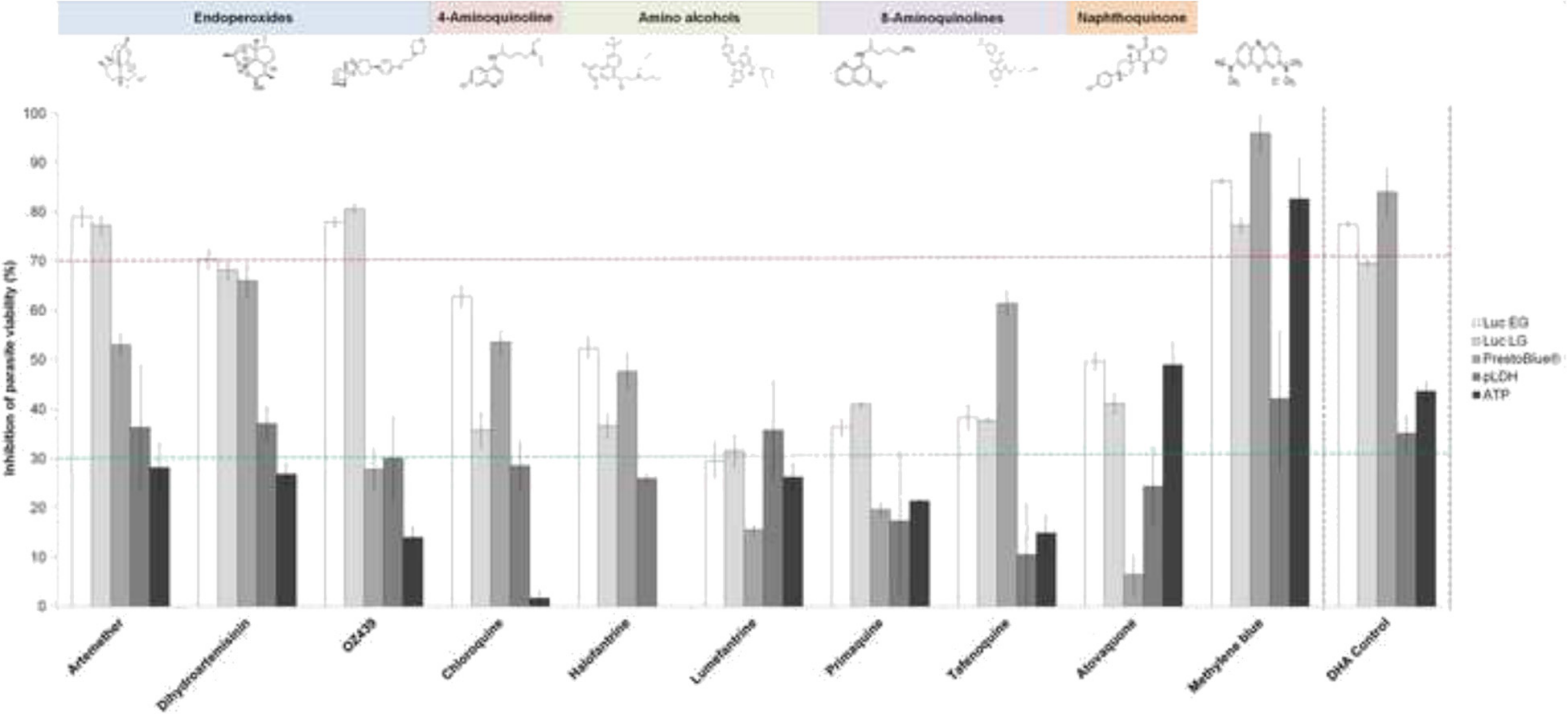
Comparative analysis of the performance of four assay platforms for gametocytocidal compounds. Mature stage IV/V gametocytes were assayed after 48 h continuous exposure to 1 μM drug in the four different assay platforms indicated (luciferase markers for both early (LucEG) and late gametocytes (LucLG)), based on the optimal conditions for each platform as previously indicated. The ATP data for halofantrine approximated zero. Data are of at least triplicate experiments, ± SEM

Taking assay platform differences into account, and relying on good intra-assay variability for each assay, the ATP, pLDH, luciferase reporter and PrestoBlue^®^ assays were compared in the context of a 10-compound set provided by MMV (Fig. 4). A single population of mature stage IV/V gametocytes were produced from *P. falciparum* (NF54) and split such that all the assays were performed in parallel on the same gametocytes. The only differences were that a proportion of these gametocytes were enriched to enable the ATP assay. Additionally, gametocytes were produced in parallel from the luciferase reporter lines. The remaining parameters for each assay were all comparable: in each case, single-point assays were performed at 1 μM drug for 48 h of continuous drug pressure for at least three replicates in each instance. Reproducibility was maintained with Z’-factors ≥0.8. The assays were performed on mature stage IV/V gametocytes (>90 % distribution) in all instances except for the luciferase reporter assay on early stage gametocytes (>80 % stage II-III parasites). To enable comparisons, significant inhibition of gametocyte viability is defined as 40–70 % inhibition, 30–40 % activity is seen as marginal, whereas no activity is defined as <30 % inhibition.

Although direct comparison of absolute inhibition values are difficult between assay platforms, similar trends were observed including comparable performance of the luciferase marker assay and the PrestoBlue^®^ assay for compounds such as DHA and methylene blue. At the concentration tested (1 μM), these compounds resulted in >70 % inhibition of parasite viability. The ATP and pLDH assays consistently did not detect the same level of inhibition of parasite viability for the endoperoxides (artemether and DHA, <50 % inhibition) compared to the other assay platforms, however, the luciferase and PrestoBlue^®^ assays detected >50 % inhibition on these compounds. Interestingly, only the luciferase reporter assay detected any activity for OZ439 (>70 % inhibition) whereas all the other assays defined this compound as inactive (<30 % inhibition). The ATP assay could not detect any inhibitory activity for chloroquine and halofantrine and may therefore be more sensitive to indicating the known inability of these compounds to inhibit gametocytes. Data from the luciferase reporter assays indicate that these compounds are indeed not highly active against mature gametocytes (30–50 % inhibition at 1 μM). The signal obtained for these compounds in the PrestoBlue^®^ assay may therefore rather reflect the inability of this assay in discriminating activity of compounds against earlier stages of gametocytes, whereas the ATP assay (working on an enriched mature stage gametocyte population) more accurately reflects these compounds’ activity. The PrestoBlue^®^ and pLDH assays seem to provide what may be falsely enhanced activity for compounds such as tafenoquine and lumefantrine, respectively. However, the PrestoBlue^®^ assay did not correlate to the other assays for Atovaquone, reporting poor activity of this compound.

## Discussion

Sustainable malaria control dictates the integration of therapeutic strategies targeting the pathogenic asexual forms as well as the transmissible sexual gametocyte forms of malaria parasites. Drug discovery is a long, arduous and expensive process, and to avoid duplication in screening libraries for gametocytocidal compounds, especially in the face of limited resources, it would be useful to compare assay results generated in different laboratories. Currently there are often discrepancies in the data, which hampers progress in this field. Two major challenges in this regard are the lack of standardization in: 1) culturing reproducible batches of pure, stage-specific gametocytes in high yield and 2) assays for the metabolically hypo-active gametocytes.

### Gametocyte production

Gametocytogenesis can be mimicked in vitro under very diverse conditions, which makes standardization difficult and also reflects our lack of knowledge of the in vivo biological process by which *P. falciparum* parasites generate gametocytes. One of the major problems with current gametocyte production protocols is that they are frequently not reproducible, and are not robust when applied in different settings. Furthermore, the inherent biological variability in different parasite populations in culture introduces additional challenges. In this study a protocol was optimized, which consistently generated high quality gametocytes in three different laboratories, confirming the robustness of the method. These gametocytes were used to evaluate and directly compare different screening assays using a set of 10 drugs supplied by MMV. The method exploited the inherent biological conversion of asexual parasites to gametocytes, confirming that not all asexual parasites will convert to gametocytes even under optimal conditions. Recently, gametocyte conversion has been shown to be under a transcriptional regulator (PfAp2G) that is activated in a stochastic manner, leading to a normally low frequency (~12.5 %) of gametocyte conversion [55]. The high conversion of approximately 33 % obtained in the current study therefore points to the direct response of the parasites to external stressors to obtain at least a doubling in gametocyte conversion in the in vitro system. The increased production of gametocytes did not change the gender ratio of approximately 4:1 females:males as has been observed in vivo [8, 11]. For gametocytes produced in vitro to be used to evaluate the gametocytocidal activity of compound libraries, it is important to monitor the quality of the parasites prior to performing the assay, including assessment of gametocyte viability and functionality (e.g. ability to form male gametes); purity (absence of asexual parasites) and stage-specificity (>90 % of stage IV/V in this study).

### Gametocytocidal screening assays

Endeavours to find transmission blocking anti-malarials are currently hindered by a lack of understanding of the basic biological processes governing gametocytogenesis in the human host, which makes an informed choice of an assay system problematic. In humans, gametocytes are suggested to be terminally differentiated and metabolically less active [32]. It is proposed that there is no replication of the gametocyte genome during development and that gametocytes are arrested in phase G_0_ of the cell cycle [56]. Nucleic acid synthetic activity is likely restricted to RNA synthesis and genetic evidence shows that gametocytes are haploid. Synthesis of RNA is reported to stop after day 6 of development, and there is no haemoglobin digestion and protein synthesis in mature gametocytes [57, 58]. Any assay for gametocytocidal activity therefore currently relies on measuring changes in metabolic status or stress responses in gametocytes, performed against the background of a metabolically ‘inactive’ cell.

Several groups have published platforms allowing the evaluation of gametocytocidal activity of a compound series. A Bill and Melinda Gates Foundation Initiative recently attempted to compare gametocyte assay platforms from various laboratories using different assay readouts (reported elsewhere). However, direct comparison of the data from these studies is fraught with difficultly due to differences in parameters such as: 1) parasite strains used; 2) different gametocyte induction protocols; 3) composition of culture medium used; 4) gametocyte isolation protocols; 5) stage of development of gametocytes; 6) assay platforms; 7) presence or absence of erythrocytes; 8) number of gametocytes per assay well; 9) panel of compounds; 10) concentration of compounds; 11) drug exposure times; 12) presentation of data, e.g. % inhibition at single concentrations or only IC_50_ values, etc.

The production of viable and functional gametocytes using a standardized, robust gametocyte production protocol was used here to overcome the limitations listed above. These gametocytes were subsequently used, for the first time, in a parallel, comparative interrogation of four different gametocytocidal assays (ATP, pLDH, PrestoBlue^®^ and the luciferase reporter) for their ability to detect gametocytocidal activity of a single set of 10 compounds from MMV. The compound set was provided blinded and only unblinded after completion of all the assays and data analysis. The parallel nature of the assays performed on the same gametocyte population uniquely allowed direct comparisons of the assay platforms in this study.

### Comparison of assays that evaluate different biological functions

The tricarboxylic acid (TCA) cycle is highly active in gametocytes, especially in females that are characterized by a rapidly expanding mitochondrion in preparation for gametogenesis [59, 60]. A recent study found 15 of 16 mRNA transcripts of mitochondrial TCA cycle enzymes upregulated in gametocytes [50]. Increased TCA cycle activity implies increased levels of cytoplasmic ATP. In the luminescent ATP assay, ATP content is used to evaluate the functional integrity of living cells, as injured/dead cells will display drastically reduced ATP levels [61]. This platform should, therefore, be reliable for the assessment of compounds for their ability to inhibit gametocytes. However, the assay requires the downstream manipulation of gametocytes (enrichment and isolation) during which gametocyte viability is compromized. ATP levels decrease by as much as 50 % within the first 16 h after enrichment, and this level steadily decreases as gametocytes are further incubated after enrichment to a total level of only 20 % remaining at 48 h, which is the usual timeframe for assaying drug effects. This then influences the number of viable gametocytes remaining in the population used to detect drug effects. Moreover, although this manipulation results in high S/N ratios due to gametocytes being enriched above background, it could contribute to higher inter-assay variability. However, the ATP assay is possibly less prone to artefacts compared to fluorescent viability assays [62].

One major advantage of the pLDH assay is that it is performed directly on parasite cultures, therefore minimising manipulation of gametocytes. pLDH has been shown to be present in the blood of malaria patients and to be a reliable marker for gametocyte viability as the enzyme is present at high levels throughout gametocytogenesis [63, 64]. pLDH catalyses the conversion of pyruvate, the final product of glycolysis, to lactate. This flux is important for the regeneration of NAD^+^, itself an essential cofactor for glycolysis. Only when increased fermentative glycolysis is possible, do cells exhibit increased proliferation through the anabolic capacity of glycolysis [65]. However, late stage gametocytes exhibit decreased expression of genes responsible for glycolysis, protein biosynthesis and haemoglobin catabolism [50]. As mentioned, terminally differentiated gametocytes make use of a canonical TCA cycle, and less glucose is metabolized by fermentation to lactate. Reduced glycolytic activity suggests that less pyruvate is converted to lactate by LDH, which might explain the relatively low S/B values obtained for the pLDH assay. A direct comparison of the pLDH activity in asexual parasites, early and late stage gametocytes will be valuable in confirmation of assay reliability. Additionally, the continued presence of pLDH activity even after parasite death has to be taken into account [34].

Redox reactive (oxidoreductive indicators) cell permeable dyes like alamarBlue^®^ and PrestoBlue^®^ have previously been reported as robust assays amenable to high-throughput screens, more sensitive than traditionally used tetrazolium dyes [31, 41]. These assays enable ease of use when screening gametocyte populations. However, care must be taken with these assay platforms in the case where compounds target the redox state of the parasite, as it may interfere with the assay readout. Moreover, compounds routinely used like methylene blue cannot be used in this assay platform due to colourimetric interference.

Despite the drawbacks of each assay, the ATP, pLDH and PrestoBlue^®^ assays are useful for assaying gametocytocidal activity of compounds on non-genetically modified lab strains as well as clinical isolates. This information will become increasingly important in drug development programmes, enabling early detection of cross-resistance or efficacy failure of lead gametocytocidal compounds. Assay cascades may also be influenced from an economic point of view, with the PrestoBlue^®^ the least expensive (~$1 per compound) followed by the pLDH and lastly the luminescence ATP and luciferase assays (up to a 7-fold increase in cost).

Compared to the assays above, the bioluminescent luciferase reporter lines enabled stage-specific gametocytocidal activity detection through endogenous luciferase production under stage-specific reporters, again with minimal interference of the luminescent signal by the compounds screened. This assay platform was robust in all cases resulting in high S/N ratios and in quantification of the internal signal. This platform has recently been further optimized to a dual-colour assay to simultaneously and quantitatively assay the viability of different stages of gametocyte populations [66].

In addition to the above, the blinded nature of the study and the fact that the assays were performed in parallel allow for a situation where assay platforms can be compared directly. The data were validated since the DHA control included in the study correlated very well for all assay platforms with the blinded DHA control included in the compound panel.

As reported [35, 38], the endoperoxides were able to equally target both the early- and late-stage gametocytes and were the most active compounds tested with IC_50_s in the nM range in some assay platforms. The equipotency of the endoperoxides to early- and late-stage gametocytes was confirmed here particularly with the luciferase reporter assay (for artemether, DHA and OZ439). These data are also comparable to the late-stage gametocytocidal activity of endoperoxides (artemether and DHA) [34]. The endoperoxides are amongst the most potent anti-malarials, fast acting and thought to act through alkylation of haem or other biomolecules [67] and requiring iron-mediated activation of the endoperoxide bridge. The ability of artemisinins particularly to oxidize cofactors of parasite flavoenzymes contributes to generating cytotoxic metabolites and reactive oxygen species, resulting in oxidative damage to cells [68, 69]. The PrestoBlue^®^ assay was able to detect comparable levels of activity to the luciferase reporter assay particularly for DHA, indicating that the oxidoreductive dye is able to measure drug response in the context of oxidative cellular stress, at least for the compounds tested here. It has however been postulated that another target for the endoperoxides may be direct interaction with PfATP6, interfering with ATP synthesis [69]. As mature gametocytes do not metabolize haemoglobin effectively, the latter may indeed be the physiological mode of action of these compounds in the sexual parasites. Interestingly, the ATP assay indicated poor activity of the endoperoxides compared to the luciferase and PrestoBlue^®^ assays, previously reported as well [21, 33]. Within the endoperoxide group, OZ439 was shown to have potent gametocytocidal activity with the luciferase reporter assay in the low nM range, however, none of the other assay platforms were able to detect this activity. This confirmation of the ability of the endoperoxides to target both immature and mature gametocytes sheds light on artemisinin-based combination therapy as transmission blocking drugs, an effect that should not solely be ascribed to the extremely rapid clearance of asexual parasites and young gametocytes. Surprisingly, the endoperoxides seem to exclusively target male gametocytes preventing male gamete formation; the exact reason for this is unclear [70]. The ATP assay has been reported to be a poor indicator of the gametocytocidal activity of endoperoxides [21, 33] and this was confirmed here with IC_50_s of 15 μM obtained for DHA compared to nanomolar IC_50_s seen with the pLDH, PrestoBlue^®^ and luciferase assays. Alternatively, gametocytes may stay viable in the presence of endoperoxides or at least maintain their ATP pool and LDH activities while some gene promoters are not as active, explaining the difference between reporter gene and metabolic readouts.

The 4-aminoquinolines (chloroquine) were confirmed to be more active against asexual parasites and early stage gametocytes, confirming their targeting of haemozoin formation in these stages of the parasite. Whilst the PrestoBlue^®^ and pLDH assays did seem to indicate some activity against late-stage parasites, the ATP assay is possibly more informative for this chemotype. Comparatively, the 8-aminoquinolines tested here (primaquine and tafenoquine) performed poorly against both early and late stage gametocytes. However, for these compounds, the ATP assay was able to detect low inhibitory activities, implying differences in the mode of action between the 4- and 8-aminoquinolines on mature gametocytes or interference of the compounds tested (primaquine and tafenoquine) with the assay platform.

The 8-aminoquinolines are known to be metabolically activated by liver enzymes, hence eliciting activity against liver stage hypnozoite forms of malaria parasites [71]. However, in the assay systems employed here, such metabolic activation is not possible but could be resolved by pre-exposure of the drugs to liver cell extracts before analysing their gametocytocidal activity. Needless to say, such metabolic activation is not considered in medium- to high-throughput screening assays of unknown compounds. However, the PrestoBlue^®^ assay seems to report ‘enhanced’ activity of particularly tafenoquine compared to the other assay systems used, but this is not the case for primaquine. Primaquine is known to have activity against liver stages of *P. falciparum*, *Plasmodium vivax* and *Plasmodium ovale* and is, therefore, of interest in transmission blocking strategies. Primaquine has been shown to be gametocytocidal against all *Plasmodium* species for late stage gametocytes through targeting the parasites’ mitochondria, but is not clinically useful against *P. falciparum* asexual stages. However, at in vitro gametocytocidal IC_50_ values of 1-15 μM [15, 72], this compound would be identified as not active, as confirmed by the data presented here (<50 % inhibition observed on all assay platforms). This discrepancy between activity observed in vivo and the lack thereof in vitro supports the notion of metabolic activation of primaquine in vivo and thus hampers the use of in vitro assay systems for this class of compounds.

The naphthoquinone atovaquone was able to inhibit ~50 % of particularly immature gametocytes with previous proposed actions including the targeting of the electron transport chain through the cytochrome b ubiquinol oxidation site [73]. Gametocytes do however have active mitochondria [8, 65] and according to the ATP levels measured, these parasites are still 50 % viable and may indicate static arrest of the gametocytes after atovaquone treatment. The PrestoBlue^®^ assay was unable to detect this inhibitory capacity at a primary screening concentration of 1 μM, which may be a concern when such assay platforms are solely used to derive chemical signatures of libraries for gametocytocidal activity [35, 42]. As this assay supposedly provides a direct readout of cellular respiration, this is either a more sensitive probe of decreased glycolysis and respiration of the parasite upon atovaquone treatment or an indication of pluri-pharmacology of atovaquone in gametocytes. When atovaquone was rescreened against the PrestoBlue^®^ assay at 10 μM, gametocytocidal activity was however noted for this compound (71 +/- 1.9 % inhibition), highlighting the need to define an optimal concentration threshold for primary screens that minimizes both false-negative and false-positive hit rates. In the case of synthetic compound library screens, the effects of false-negative losses may be lessened if related compounds of the same basic scaffold are identified to be active [74].

## Conclusions

The standardized protocol produced a reproducible, high gametocytaemia and these parasites were viable, functional and could be used in gametocytocidal assays. An important point that emerged from this study is that unlike asexual parasite assays measuring parasite proliferation, greater variability in end-point readout exists between gametocytocidal assays that interrogate different parasite biological functions. Drug mode of action is likely to be an important factor in this outcome. This suggests that compounds targeting a specific biological pathway may fail in one assay, but be active when evaluated in a different assay. Reliance on a single assay platform to screen different pharmacophores may therefore result in false negative results. However, an assay that has been demonstrated to be sensitive to a particular pharmacophore/mode-of-action may be used to screen compounds of the same series and additionally provide data that is informative from a biological perspective and may provide indications of the drug mode of action. This does highlight the care that must be taken when screening broad chemotypes with a single assay platform against gametocytes for which the biology is not clearly understood.

## Additional files

Additional file 1: Table S1. Primer sequences for qPCR.

Additional file 2: **Additional gametocyte viability indicators.**

Additional file 3: **Gametocytodical assay metrics.**

## Abbreviations

ACT: Artemisinin-based combination therapy
APAD: 3-acetylpyridine adenine dinucleotide
CV: Coefficient of variation
DHA: Dihydroartemisinin
G6PD: Glucose-6-phosphate dehydrogenase
IRS: Indoor residual spraying
ITNs: Insecticide treated bednets
MMV: Medicines for Malaria Venture
MB: Methylene blue
NAG: N-acetyl glucosamine
NBT: Nitro blue tetrazoliumchloride
NPPs: New permeation pathways
pLDH: Parasite lactate dehydrogenase
PES: Phenazine ethosulphate
XA: Xanthurenic acid
S/N: Signal to noise ratio
S/B: Signal to background ratio

## References

[1] WHO (2013). World Malaria Report.

[2] Burrows JN, Wells TN, Chibale K (2011). The state of the art in anti-malarial drug discovery and development. Curr Top Med Chem.

[3] Burrows JN, Leroy D, Lotharius J, Waterson D (2011). Challenges in antimalarial drug discovery. Future Med Chem.

[4] Sinden RE, Sherman IW (1998). Malaria. Parasite Biology, Pathogenesis, and Protection.

[5] Dixon MW, Thompson J, Gardiner DL, Trenholme KR (2008). Sex in Plasmodium: a sign of commitment. Trends Parasitol.

[6] Kuehn A, Pradel G (2010). The Coming-Out of Malaria Gametocytes. J Biomed Biotechnol.

[7] Pradel G (2007). Proteins of the malaria parasite sexual stages: expression, function and potential for transmission blocking strategies. Parasitology.

[8] Baker DA (2010). Malaria gametocytogenesis. Mol Biochem Parasitol.

[9] Silvestrini F, Alano P, Williams JL (2000). Commitment to the production of male and female gametocytes in the human malaria parasite *Plasmodium falciparum*. Parasitology.

[10] Smith TG, Lourenco P, Carter R, Walliker D, Ranford-Cartwright LC (2000). Commitment to sexual differentiation in the human malaria parasite, *Plasmodium falciparum*. Parasitology.

[11] Paul REL, Brey PT, Robert V (2002). *Plasmodium* sex determination and transmission to mosquitoes. Trends Parasitol.

[12] Trager W (2005). What triggers the gametocyte pathway in *Plasmodium falciparum*?. Trends Parasitol.

[13] Wells TN, Alonso PL, Gutteridge WE (2009). New medicines to improve control and contribute to the eradication of malaria. Nat Rev Drug Discov.

[14] Sinden RE (2010). A biologist’s perspective on malaria vaccine development. Hum Vaccin.

[15] Lucantoni L, Avery V (2012). Whole-cell *in vitro* screening for gametocytocidal compounds. Future Med Chem.

[16] Saliba KS, Jacobs-Lorena M (2013). Production of Plasmodium falciparum gametocytes *in vitro*. Methods Mol Biol.

[17] Dixon MW, Peatey CL, Gardiner DL, Trenholme KR (2009). A green fluorescent protein-based assay for determining gametocyte production in *Plasmodium falciparum*. Mol Biochem Parasitol.

[18] Fivelman QL, McRobert L, Sharp S, Taylor CJ, Saeed M, Swales CA (2007). Improved synchronous production of *Plasmodium falciparum* gametocytes *in vitro*. Mol Biochem Parasitol.

[19] Roncales M, Vidal-Mas J, Leroy D, Herreros E (2012). Comparison and optimization of different methods for the *in vitro* production of Plasmodium falciparum Gametocytes. J Parasitol Res.

[20] Delves M, Plouffe D, Scheurer C, Meister S, Wittlin S, Winzeler EA (2012). The activities of current antimalarial drugs on the life cycle stages of Plasmodium: a comparative study with human and rodent parasites. PLoS Med.

[21] Peatey CL, Leroy D, Gardiner DL, Trenholme KR (2012). Anti-malarial drugs: how effective are they against *Plasmodium falciparum* gametocytes?. Malar J.

[22] Puta C, Manyando C (1997). Enhanced gametocyte production in Fansidar-treated *Plasmodium falciparum* malaria patients: implications for malaria transmission control programmes. Trop Med Int Health.

[23] Smalley ME, Brown J (1981). *Plasmodium falciparum* gametocytogenesis stimulated by lymphocytes and serum from infected Gambian children. Trans R Soc Trop Med Hyg.

[24] Lingnau A, Margos G, Maier WA, Seitz HM (1993). The effects of hormones on the gametocytogenesis of *Plasmodium falciparum in vitro*. Appl Parasitol.

[25] Trager W, Gill GS, Lawrence C, Nagel RL (1999). *Plasmodium falciparum*: enhanced gametocyte formation *in vitro* in reticulocyte-rich blood. Exp Parasitol.

[26] Ono T, Ohnishi Y, Nagamune K, Kano M (1993). Gametocytogenesis induction by Berenil in cultured *Plasmodium falciparum*. Exp Parasitol.

[27] Salcedo-Sora JE, Caamano-Gutierrez E, Ward SA, Biagini GA (2014). The proliferating cell hypothesis: a metabolic framework for *Plasmodium* growth and development. Trends Parasitol.

[28] Ono T, Nakabayashi T (1990). Gametocytogenesis induction by ammonium compounds in cultured *Plasmodium falciparum*. Int J Parasitol.

[29] Barnes KI, Little F, Mabuza A, Mngomezulu N, Govere J, Durrheim D (2008). Increased gametocytemia after treatment: an early parasitological indicator of emerging sulfadoxine-pyrimethamine resistance in falciparum malaria. J Infect Dis.

[30] Sowunmi A, Balogun ST, Gbotosho GO, Happi CT (2008). *Plasmodium falciparum* gametocyte sex ratios in children with acute, symptomatic, uncomplicated infections treated with amodiaquine. Malar J.

[31] Tanaka TQ, Williamson KC (2011). A malaria gametocytocidal assay using oxidoreduction indicator, alamarBlue. Mol Biochem Parasitol.

[32] Peatey CL, Spicer TP, Hodder PS, Trenholme KR, Gardiner DL (2011). A high-throughput assay for the identification of drugs against late-stage *Plasmodium falciparum* gametocytes. Mol Biochem Parasitol.

[33] Lelievre J, Almela MJ, Lozano S, Miguel C, Franco V, Leroy D (2012). Activity of clinically relevant antimalarial drugs on *Plasmodium falciparum* mature gametocytes in an ATP bioluminescence “transmission blocking” assay. PLoS One.

[34] D’Alessandro S, Silvestrini F, Dechering K, Corbett Y, Parapini S, Timmerman M (2013). A *Plasmodium falciparum* screening assay for anti-gametocyte drugs based on parasite lactate dehydrogenase detection. J Antimicrob Chemother.

[35] Bolscher JM, Koolen KM, van Gemert GJ, van de Vegte-Bolmer MG, Bousema T, Leroy D (2015). A combination of new screening assays for prioritization of transmission-blocking antimalarials reveals distinct dynamics of marketed and experimental drugs. J Antimicrob Chemother.

[36] Peatey CL, Skinner-Adams TS, Dixon MW, McCarthy JS, Gardiner DL, Trenholme KR (2009). Effect of antimalarial drugs on *Plasmodium falciparum* gametocytes. J Infect Dis.

[37] Adjalley SH, Johnston GL, Li T, Eastman RT, Ekland EH (2011). Quantitative assessment of *Plasmodium falciparum* sexual development reveals potent transmission-blocking activity by methylene blue. Proc Natl Acad Sci U S A.

[38] Duffy S, Avery VM (2013). Identification of inhibitors of *Plasmodium falciparum* gametocyte development. Malar J.

[39] Chevalley S, Coste A, Lopez A, Pipy B, Valentin A (2010). Flow cytometry for the evaluation of anti-plasmodial activity of drugs on *Plasmodium falciparum* gametocytes. Malar J.

[40] Lucantoni L, Duffy S, Adjalley SH, Fidock DA, Avery V (2013). Identification of MMV malaria box inhibitors of *Plasmodium falciparum* early-stage gametocytes using a luciferase-based high- throughput assay. Antimicrob Agents Chemother.

[41] Tanaka TQ, Dehdashti SJ, Nguyen DT, McKew JC, Zheng W, Williamson KC (2013). A quantitative high throughput assay for identifying gametocytocidal compounds. Mol Biochem Parasitol.

[42] Sun W, Tanaka TQ, Magle CT, Huang W, Southall N, Huang R (2014). Chemical signatures and new drug targets for gametocytocidal drug development. Sci Rep.

[43] Trager W, Jensen JB (1976). Human malaria parasites in continous culture. Science.

[44] Allen RJ, Kirk K (2010). *Plasmodium falciparum* culture: the benefits of shaking. Mol Biochem Parasitol.

[45] Carter R, Ranford-Cartwright LC, Alano P (1993). The culture and preparation of gametocytes of *Plasmodium falciparum* for immunochemical, molecular, and mosquito infectivity studies. Protocols in Molecular Parasitology.

[46] Makler MT, Hinrichs DJ (1993). Measurement of the lactate dehydrogenase activity of *Plasmodium falciparum* as an assessment of parasitaemia. Am Trop Med Hyg.

[47] Zhang JH, Chung TD, Oldenburg KR (1999). A simple statistical parameter for use in evaluation and validation of high throughput screening assays. J Biomol Screen.

[48] Iversen PW, Beck B, Chen Y, Dere W, Devanarayan V, Eastwood BJ, et al. HTS assay validation. In: Sittampalam GS G-EN, Arkin M, et al., editors. Assay Guidance Manual. Bethesda (MD), USA: Eli Lilly & Co and National Centre for Advancing Translational Sciences; 2012.

[49] Hawking F, Wilson ME, Gammage K (1971). Evidence for cyclic development and short-lived maturity in the gametocytes of *Plasmodium falciparum*. Trans R Soc Trop Med Hyg.

[50] Young JA, Fivelman QL, Blair PL, de la Vega P, Le Roch KG, Zhou Y (2005). The *Plasmodium falciparum* sexual development transcriptome: a microarray analysis using ontology-based pattern identification. Mol Biochem Parasitol.

[51] Zhang L, Albarède S, Dumont G, Van Campenhout C, Libeer J, Albert A (2010). The multivariate coefficient of variation for comparing serum protein electrophoresis techniques in External Quality Assessment schemes. Accreditation and Quality Assurance.

[52] Martín-Navarro CM, López-Arencibia A, Sifaoui I, Reyes-Batlle M, Cabello-Vílchez AM, Maciver S, et al. PrestoBlue and AlamarBlue are equally useful as agents to determine the viability of *Acanthamoeba* trophozoites. Exp Parasitol. 2014;1–4. 10.1016/j.exppara.2014.03.024 24703973

[53] Life Technologies Corporation (2010). PrestoBlue® Cell Viability Reagent.

[54] Eksi S, Suri A, Williamson KC (2008). Sex- and stage-specific reporter gene expression in *Plasmodium falciparum*. Mol Biochem Parasitol.

[55] Kafsack BF, Rovira-Graells N, Clark TG, Bancells C, Crowley VM, Campino SG (2014). A transcriptional switch underlies commitment to sexual development in malaria parasites. Nature.

[56] Raabe AC, Billker O, Vial HJ, Wengelnik K (2009). Quantitative assessment of DNA replication to monitor microgametogenesis in *Plasmodium berghei*. Mol Biochem Parasitol.

[57] Canning EU, Sinden RE (1975). Nuclear organisation in gametocytes of *Plasmodium* and hepatocystis: a cytochemical study. Z Parasitenkd.

[58] Sinden RE, Canning EU, Bray RS, Smalley ME (1978). Gametocyte and gamete development in *Plasmodium falciparum*. Proc R Soc Lond B Biol Sci.

[59] Khan SM, Franke-Fayard B, Mair GR (2005). Proteome analysis of separated male and female gametocytes reveals novel sex-specific *Plasmodium* biology. Cell.

[60] Okamoto N, Spurck TP, Goodman CD, McFadden GI (2009). Apicoplast and mitochondrion in gametocytogenesis of *Plasmodium falciparum*. Eukaryot Cell.

[61] Crouch SP, Kozlowski R, Slater KJ, Fletcher J (1993). The use of ATP bioluminescence as a measure of cell proliferation and cytotoxicity. J Immunol Methods.

[62] Riss TL, Moravec RA, Niles AL, Benink HA, Worzella TJ, Minor L, Sittampalam GS G-EN, Arkin M (2012). Cell viability assays. Assay Guidance Manual.

[63] Oduola AM, Omitowoju GO, Sowunmi A (1997). Plasmodium falciparum: evaluation of lactate dehydrogenase in monitoring therapeutic responses to standard antimalarial drugs in Nigeria. Exp Parasitol.

[64] Mueller I, Betuela I, Ginny M (2007). The sensitivity of the OptiMAL rapid diagnostic test to the presence of *Plasmodium falciparum* gametocytes compromises its ability to monitor treatment outcomes in an area of Papua New Guinea in which malaria is endemic. J Clin Microbiol.

[65] MacRae JI, Dixon MWA, Dearnley MK, Chua HH, Chambers JM, Kenny S (2013). Mitochondrial metabolism of sexual and asexual blood stages of the malaria parasite *Plasmodium falciparum*. BMC Biol.

[66] Cevenini L, Camarda G, Michelini E, Siciliano G, Calabretta MM, Bona R (2014). Multicolor bioluminescence boosts malaria research: quantitative dual-color assay and single-cell imaging in *Plasmodium falciparum* parasites. Anal Chem.

[67] O’Neill PM, Barton VE, Ward SA (2010). The molecular mechanism of action of artemisinin–the debate continues. Molecules.

[68] Haynes RK, Cheu KW, Chan HW, Wong HN, Li KY, Tang MM (2012). Interactions between artemisinins and other antimalarial drugs in relation to the cofactor model–a unifying proposal for drug action. ChemMedChem.

[69] Klonis N, Crespo-Ortiz MP, Bottova I, Abu-Bakar N, Kenny S, Rosenthal PJ (2011). Artemisinin activity against *Plasmodium falciparum* requires hemoglobin uptake and digestion. Proc Natl Acad Sci U S A.

[70] Delves MJ, Ruecker A, Straschil U, Lelievre J, Marques S, Lopez-Barragan MJ (2013). Male and female *Plasmodium falciparum* mature gametocytes show different responses to antimalarial drugs. Antimicrob Agents Chemother.

[71] Wells TN, Burrows JN, Baird JK (2010). Targeting the hypnozoite reservoir of Plasmodium vivax: the hidden obstacle to malaria elimination. Trends Parasitol.

[72] Dechy-Cabaret O, Benoit-Vical F (2013). Effects of antimalarial molecules on the gametocyte stage of *Plasmodium falciparum*: the debate. J Med Chem.

[73] Fowler RE, Billingsley PF, Pudney M, Sinden RE (1994). Inhibitory action of the anti-malarial compound atovaquone (566C80) against *Plasmodium berghei* ANKA in the mosquito, *Anopheles stephensi*. Parasitology.

[74] Malo N, Hanley JA, Cerquozzi S, Pelletier J, Nadon R (2006). Statistical practice in high-throughput screening data analysis. Nat Biotechnol.

