# Supplementary material for: Nowhere to hide: interrogating different metabolic parameters of Plasmodium falciparum gametocytes in a transmission blocking drug discovery pipeline towards malaria elimination
Source: Malar J. 2015 May 22;14:213. doi: 10.1186/s12936-015-0718-z (PMC4449569; doi:10.1186/s12936-015-0718-z)
Supplement: Supplementary file 1 — Primer sequences for qPCR. [file 12936_2015_718_MOESM1_ESM.docx]

**Table S1. Primer sequences for qPCR**

| **Transcript identifier** | **Oligonucleotide sequence (5’ – 3’)**  **forward and reverse** |
| --- | --- |
| Pf3D7_0831400 | 5'-TAAGAAATGAAATTATGGATG-3'  5'-CCTCGTTTATATATTTACCTG C-3 |
| Pf3D7_1250100 | 5’-GCGACAGATGAACAACAGGT-3’  5’-CACTTGGTTTTGATTATCTCCC-3’ |
| Pf3D7_0406200 | 5’-TGCTTATATTCTTCGCTTTTGC-3’  5’-TAGTCCACCTTGATTAGGTCCA-3’ |
| Pf3D7_1038400 | 5’-ATCATTTACGCTTTAGAGG-3’  5’-CTTCTTATGTTTCGGTTTTA-3’ |
| Pf3D7_0209000 | 5’-CCACTTATTTTATTATTCCCAC-3’  5’-GTTCTTTGTTTTATTTTTACGG-3’ |
| Pf3D7_1228300 | 5’-GTGCTTTACAGTGTTTGGGA-3’  5’-TTAATACATCATTCGGATGAGC-3’ |
| Pf3D7_1302100 | 5’-CCTCGTATTAGAAAAGTTGGG-3’  5’-ATCTATTTTGATTCTTGCGAAC-3’ |
| Pf3D7_0617900 | 5'-CCCCAAGAAAACAACTCGC-3'  5'-AAAGCAACAGTTCCTGGACG-3 |
| Pf3D7_0625100 | 5'-TTACCCGCAACATTAGAAAC-3'  5'-TTGTTGAAAAAGAATATGTCCTG-3 |
| Pf3D7_0621400 | 5’-GAAAAAGAGGACGATGGTT-3’  5’-CATTAAAAACGGGTTGATCT-3’ |
| Pf3D7_0501200 | 5'-TACGCCAAGAATCAAGAAC-3'  5'-GGGTCAAGTGCGTAACTAA-3' |
| Pf3D7_1477300 | 5'-AGTGAAACTGAACCACCG-3'  5'-GATTTTCTTCCGTCAAC-3' |
| Pf3D7_0500800 | 5'-ATGAAATAATTCGTGCGA G-3'  5'-CTGTAACAACCGAACCCC-3' |
| Pf3D7_0525800 | 5'-GTTCCAGAAGTTAATTGCC-3'  5'-GCAAGACTTATGGTTTGG-3' |
| Pf3D7_1035800 | 5'-AAATTCGGATTCTAATGTG-3'  5'-CAACTCATCTTCTTCGC-3' |
| Pf3D7_0309100 | 5'-CTCAAAAAGACTCGTACAAT-3'  5'-GCTGAACATTAATCATAGC-3' |
| Pf3D7_0717500 | 5'-CCTTTCAACGGGTCGAACG-3'  5'-GCGTCCCTTGCCGATATCC-3' |
| Pf3D7_0411700 | 5'-TGGAAATATTAAATTCGAGCG-3'  5'-TGGCTTTATGGAACTTGTC-3' |
