# Supplementary material for: Nowhere to hide: interrogating different metabolic parameters of Plasmodium falciparum gametocytes in a transmission blocking drug discovery pipeline towards malaria elimination
Source: Malar J. 2015 May 22;14:213. doi: 10.1186/s12936-015-0718-z (PMC4449569; doi:10.1186/s12936-015-0718-z)
Supplement: Supplementary file 2 — Additional gametocyte viability indicators. [file 12936_2015_718_MOESM2_ESM.docx]

**Additional File 2: Additional gametocyte viability indicators**


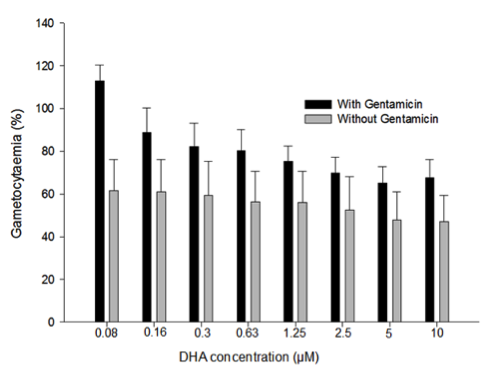

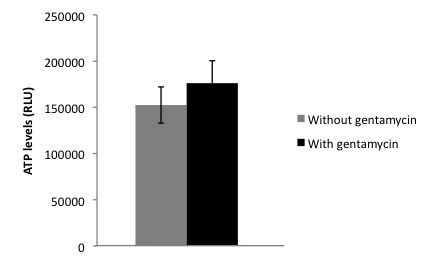


A

B

**Figure S1:** Influence of gentamycin on gametocyte viability and drug assays. (A) Gametocyte viability measured as a factor of ATP production in relative light units (RLU) in gametocytes grown either in the presence or absence of gentamycin. (B) Dose-response curve for DHA on gametocytes in the presence and absence of gentamycin.


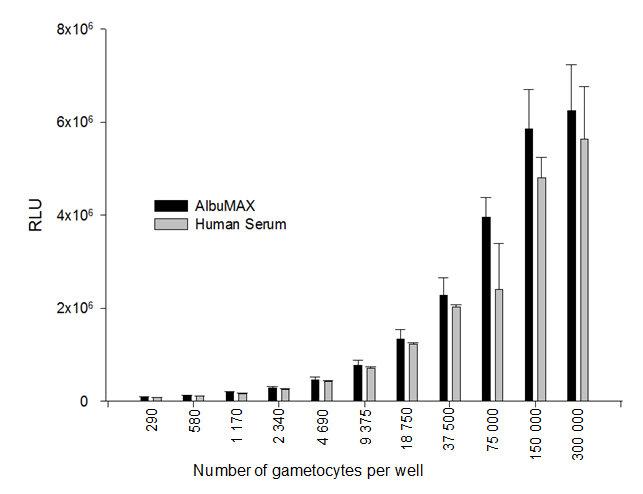


**Figure S2:** Viability of gametocytes produced under optimal conditions as evaluated by ATP production.
