# Supplementary material for: Nowhere to hide: interrogating different metabolic parameters of Plasmodium falciparum gametocytes in a transmission blocking drug discovery pipeline towards malaria elimination
Source: Malar J. 2015 May 22;14:213. doi: 10.1186/s12936-015-0718-z (PMC4449569; doi:10.1186/s12936-015-0718-z)
Supplement: Supplementary file 3 — Gametocytodical assay metrics. [file 12936_2015_718_MOESM3_ESM.docx]

**Additional File 3: Gametocytodical assay metrics**

**ATP assay**


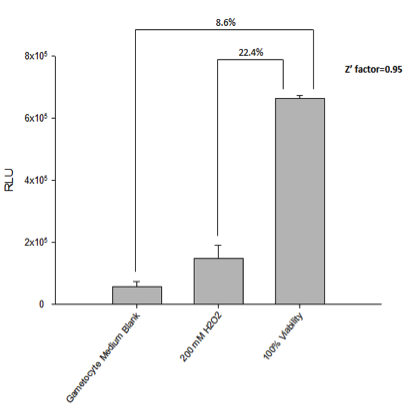

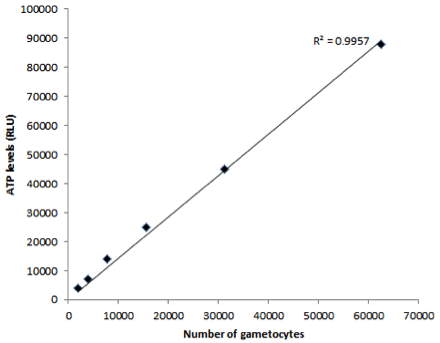


A

B

**Figure S3.** ATP assay evaluation (24 h assay). (A) Signal to noise and signal to background values for the ATP luminescence readout. (B) Linearity of ATP luminescence readout compared to number of isolated gametocytes. Data are from three independent biological repeats, each performed in triplicate, ± S.E.

**pLHD assay**


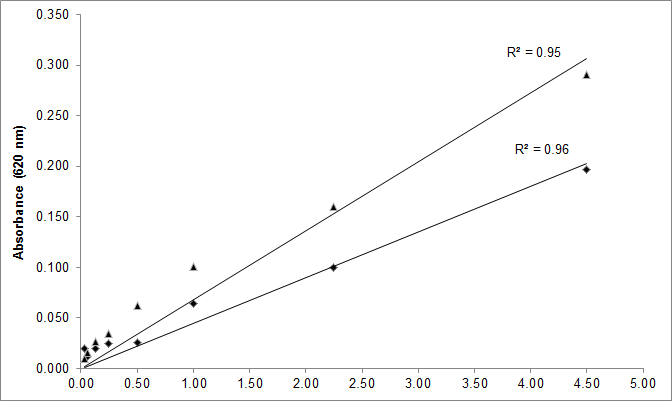


A

B

**Figure S4.** **pLDH assay evaluation** (A) Signal to noise and signal to background values for the pLDH readout. (B) Linearity of absorbance (620 nm) readout compared to gametocytaemia, at 0.5% haematocrit (triangles) and 1.0% haematocrit (squares). Data are from three independent biological repeats, each performed in triplicate, ± S.E

**
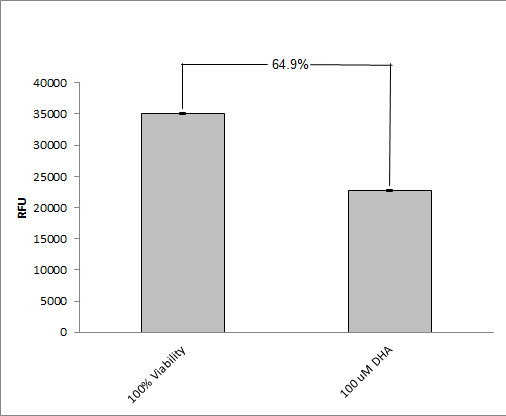

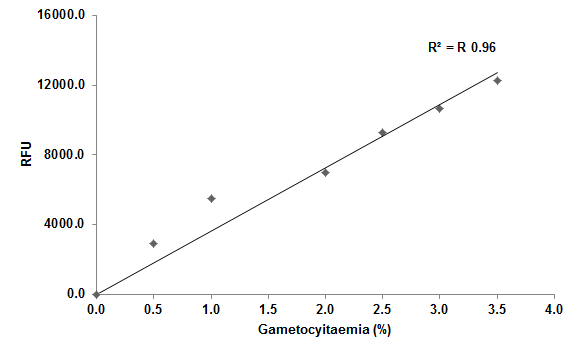
PrestoBlue assay**

A

B

**Figure S5.** PrestoBlue assay evaluation. (A) Signal to noise and signal to background values for the fluorescence readout. (B) Linearity of fluorescence readout compared to gametocytaemia. Data are from three independent biological repeats, each performed in triplicate, ± SEM.

**Bioluminescence luciferase reporter assay**

**
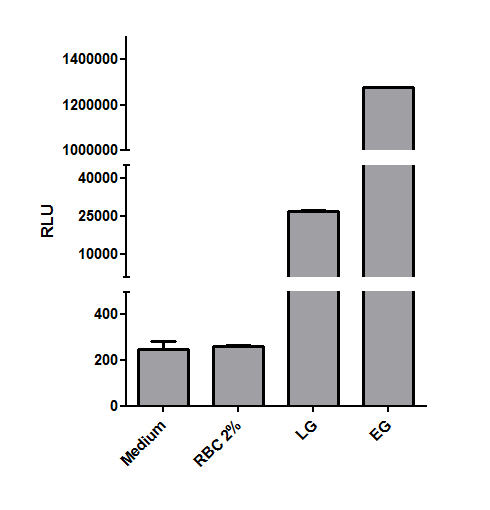
**

A

B


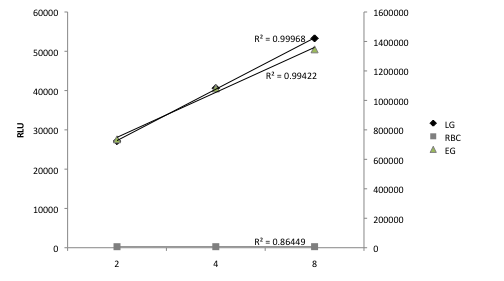


**Figure S6.** Luciferase reporter assay evaluation for early (EG) and late (LG) stage gametocytes. (A) Signal to noise and signal to background values for the luminescence readout. (B) Linearity of luminescence readout compared to gametocytaemia. Data are from three independent biological repeats, each performed in triplicate, ± S.E.
